# Prehospital analgesia for trauma-related pain by paramedics: a comparative retrospective observational study of paracetamol, nalbuphine plus paracetamol, and piritramide

**DOI:** 10.1186/s13049-025-01470-8

**Published:** 2025-10-02

**Authors:** Jonas Lohmann, Martin Deicke, Marvin Deslandes, Julia Johanna Grannemann, Lydia Johnson Kolaparambil Varghese, Jochen Hinkelbein, Annika Hoyer, Matthias Kalmbach, Thomas Plappert, Bernd Strickmann, Mathini Vaseekaran, Tobias Vollmer, Gerrit Jansen

**Affiliations:** 1 Medical School OWL, Bielefeld University, Universitätsstraße 25, 33615 Bielefeld, Germany; 2 Emergency Medical Service, County of Osnabrueck, Am Schölerberg 1, 49082 Osnabrueck, Germany; 3 Department of Anesthesiology and Operative Intensive Care Medicine, Hospital of Osnabrueck, Am Finkenhügel 1, 49076 Osnabrueck, Germany; 4 University Department of Anesthesiology, Intensive Care Medicine, Emergency Medicine and Pain Medicine, Johannes Wesling Klinikum Minden, Ruhr University Bochum, Hans- Nolte-Straße 1, 32429 Minden, Germany; 5 Emergency Medical Services, City and District of Guetersloh, Herzebrocker Strasse 140, 33324 Guetersloh, Germany; 6 Medical School OWL, Biostatistics and Medical Biometry, Bielefeld University, Universitätsstraße 25, 33615 Bielefeld, Germany; 7 Emergency Medical Services, City and District of Fulda, Otfrid-von-Weißenburg-Str. 3, 36043 Fulda, Germany; 8 Department of Anesthesiology, Intensive Care Medicine and Emergency Medicine, Hospital of Fulda, Pacelliallee 4, 36043 Fulda, Germany; 9 Emergency Medical Services of the Order of Malta, Region Hesse, Schmidtstrasse 67, 60326 Frankfurt/Main, Germany

**Keywords:** Analgesia, Opioid, Paramedic, Nalbuphine, Trauma

## Abstract

**Background:**

In this retrospective observational study, we compared the efficacy and safety of paracetamol compared with those of nalbuphine plus paracetamol and piritramide in prehospital analgesia for trauma-related pain administered by paramedics.

**Methods:**

All prehospital analgesia for trauma-related pain administered by paramedics in the emergency medical services of the districts of Fulda (piritramide) and Guetersloh (paracetamol, nalbuphine plus paracetamol) was analyzed based on electronic medical records. Primary endpoint was effective analgesia defined as a numeric rating scale (NRS) score ≤ 4 at hospital admission. Safety was evaluated based on the occurrence of drug-related complications (nausea/vomiting, antiemetic use, reduced level of consciousness, respiratory or hemodynamic insufficiency).

**Results:**

A total of 1,295 interventions (district of Guetersloh: *n* = 784 [60.5%], district of Fulda: *n* = 511 [39.5%]) were analyzed. Paracetamol: *n* = 291 [22.5%], initial NRS: 6.8 ± 1.4, final NRS: 4.0 ± 1.9. Nalbuphine plus paracetamol: *n* = 493 [38.1%], initial NRS: 8.2 ± 1.3, final NRS: 3.6 ± 1.9. Piritramide: *n* = 511 [39.5%], initial NRS: 8.3 ± 1.1, final NRS: 4.3 ± 1.5. The likelihood of achieving NRS ≤ 4 at hospital admission was highest in the nalbuphine plus paracetamol group (odds ratio (OR): 3.25, 95% confidence interval (95% CI): 2.46–4.30) and lower in the piritramide (OR: 0.31, 95% CI: 0.23–0.41) and paracetamol (OR: 0.32, 95% CI: 0.23–0.45) groups. The comparison between piritramide (*n* = 30 [5.9%]) and nalbuphine plus paracetamol (*n* = 34 [7.7%]) did not reveal evidence for differences in the occurrence of drug-related complications (OR: 0.75, 95% CI: 0.45–1.24).

**Conclusions:**

Analgesia with nalbuphine plus paracetamol for trauma patients administered by paramedics were more effective in reducing pain to NRS ≤ 4 at hospital admission compared to monotherapy with paracetamol or piritramide, with a comparable safety profile.

## Introduction

Acute pain resulting from traumatic injuries is a common reason for emergency medical services (EMS) activation [1–5]. The heterogeneity of trauma mechanisms, the potentially critical patient conditions, and operational challenges make effective pain management particularly difficult [2]. Although various analgesia concepts have been established, the incidence of oligoanalgesia remains unsatisfactorily high. This underscores the need for potent analgesics, including µ-opioid receptor agonists alone or in combination with non-opioid analgesics and the analgosedative (es-)ketamine.

Recent amendments to Germany’s Narcotics Act (Gesetz über den Verkehr mit Betäubungsmitteln; Betäubungsmittelgesetz - BtMG) now permit paramedics to administer opioid analgesics when required to avert significant risk to patients’ health or to alleviate severe symptoms [6]. Against the backdrop of a growing shortage of prehospital emergency physicians, this concept has the potential to improve patient care. Nevertheless, its use by paramedics is debated critically because of potentially life-threatening adverse effects, including respiratory and hemodynamic insufficiency.

While potent µ-opioids such as fentanyl and morphine have been evaluated in studies and are therefore frequently used internationally, the administration of piritramide by paramedics is common in some EMS districts in Germany [4]. Piritramide is a long-acting µ-opioid receptor agonist used for severe to very severe postoperative and trauma-related pain, primarily in inpatient care, in Germany, Austria, Belgium and the Netherlands. Its onset of action following intravenous administration is 15–20 min, with a peak effect after 45 min and a duration of action of 4–6 h. Its adverse effects are very similar to those of morphine. Although piritramide, despite occasionally causing bradycardia and hypotension, is considered less likely to cause circulatory depression and is also suitable for patients with renal insufficiency due to hepatic metabolization [7].

Nalbuphine, a partial µ-antagonist and k-agonist, may offer a safer alternative for paramedic use. With comparable analgesic efficacy to morphine and piritramide, it acts rapidly with an onset of action of 2–3 min, a peak effect after approximately 10 min and a duration of 3–6 h. Its ceiling effect reduces the risk of respiratory or circulatory depression, making it safer for use by paramedics [8–12].

This retrospective, multicenter observational study evaluates the efficacy and safety of nalbuphine plus paracetamol, piritramide and paracetamol for prehospital analgesia of trauma-related pain administered by paramedics to obtain comparative data and contribute to improving prehospital analgesia.

## Methods

### Study design and setting

This study is a retrospective multicentric observation cohort analysis conducted in two regional EMS districts in Germany: Fulda and Guetersloh on patients with trauma-related pain from previously published data [8]. The Fulda district (about 223,500 residents) used piritramide as opioid analgesia in the period 01/01/2018–05/31/2023, whereas Guetersloh (about 364,000 residents) implemented either paracetamol monotherapy or a combination of nalbuphine plus paracetamol in the period 01/01/2020–06/30/2022. Both districts are rural areas. The study was approved by the Ethics Committee of the Westphalia-Lippe Medical Association on 09.02.2022 (Ref. 2022-031-f-S) [7, 8].

### Inclusion and exclusion criteria

All intravenous analgesia administered by paramedics for patients aged ≥ 18 years with trauma-related pain were included. Patients < 18 years of age, analgesia for non-trauma-related pain, pregnant patients, and incompletely documented interventions were excluded. Also excluded were patients with contraindication for the analgesics (paracetamol: known allergy/intolerance, liver dysfunction, severe renal insufficiency, dialysis, pregnancy, known glucose‒6‒phosphate dehydrogenase deficiency, and blood formation disorders; nalbuphine: known allergy/intolerance, pregnancy, reduced level of consciousness, severe liver insufficiency and kidney insufficiency, long-term treatment with opioids; piritramide: Glasgow Coma Scale score (GCS) < 12, heart rate < 50/min, respiratory rate < 10/min, systolic blood pressure < 100 mmHg and SpO_2_ < 90%, presence of severe obstructive pulmonary disease).

### Analgesia procedure

After initial trauma care following the ABCDE approach (Airway, Breathing, Circulation, Disability, Exposure – an assessment and treatment protocol for focusing on the most life-threatening clinical problems), a structured medical history was taken, and pain intensity was assessed via the numeric rating scale (NRS 0–10). Non-medication analgesic measures (positioning, splinting, cooling) were applied, and basic monitoring was established (electrocardiogram, noninvasive blood pressure, SpO_2_ per pulse oximetry). For pain intensity NRS scores ≥ 4 and following the patient’s informed verbal consent, peripheral venous access was established, and analgesia was administered per local protocols.

### Analgesia with paracetamol or nalbuphine plus paracetamol in the district of Guetersloh EMS

The paramedics administered 15 mg/kg intravenous paracetamol up to a maximum dose of 1000 mg for NRS scores ranging from 4 to 7. If ineffective (NRS ≥ 4 after 5-10 min), intravenous nalbuphine was added, with an initial dose of 0.2 mg/kg nalbuphine intravenously (0.1 mg/kg for patients aged ≥ 65). If the pain persisted, a repeat dose of 0.1 mg/kg of nalbuphine could was administered once (0.1 mg/kg twice for patients aged ≤ 65) after at least two minutes up to a total dose of 20 mg. For NRS scores ranging from 8 to 10, nalbuphine plus paracetamol was administered primarily.

### Analgesia with piritramide in the district of Fulda EMS

Paramedics in the district of Fulda administered 0.06 mg/kg intravenous piritramide, with repeat doses every 5–6 min, up to a total dose of 15 mg, in case of insufficient pain relief.

The recorded data included the date of intervention, patient age, sex, location of injury (head/neck, spine, thorax, pelvis, upper extremity, lower extremity, multiple injuries, unspecified), NRS score at the beginning and end of the mission, change in pain intensity over the duration of the mission (∆-NRS), administered analgesics and their doses. Drug-related complications (nausea/vomiting, administered drug antiemesis, respiratory complications (drops in satiety, apnea/bradypnea, need for additional oxygen administration, assisted and/or controlled ventilation), hemodynamic complications (systolic blood pressure < 100 mmHg), and vigilance reduction (GCS ≤ 14)) were also recorded.

### Outcomes

The primary endpoint was effective analgesia, defined as an NRS score ≤ 4 at hospital admission [13]. The secondary endpoint was the occurrence of drug-related complications as an indicator of drug safety.

### Statistical analysis

Logistic regression was performed to analyze the primary endpoint. Covariates were the analgesia concept used (nalbuphine plus paracetamol vs. paracetamol vs. piritramide; exposure), age, sex and initial NRS score. To analyze the secondary endpoint “occurrence of at least one complication”, logistic regression with covariates analgesia concepts, age and sex was performed. The results of the logistic regressions are presented as odds ratios (ORs) with corresponding 95% confidence intervals (95% CIs) and *p*-values. All analyses were performed using SAS 9.4 (SAS Institute Inc., Cary, NC).

## Results

### Study population

**Fig. 1:**
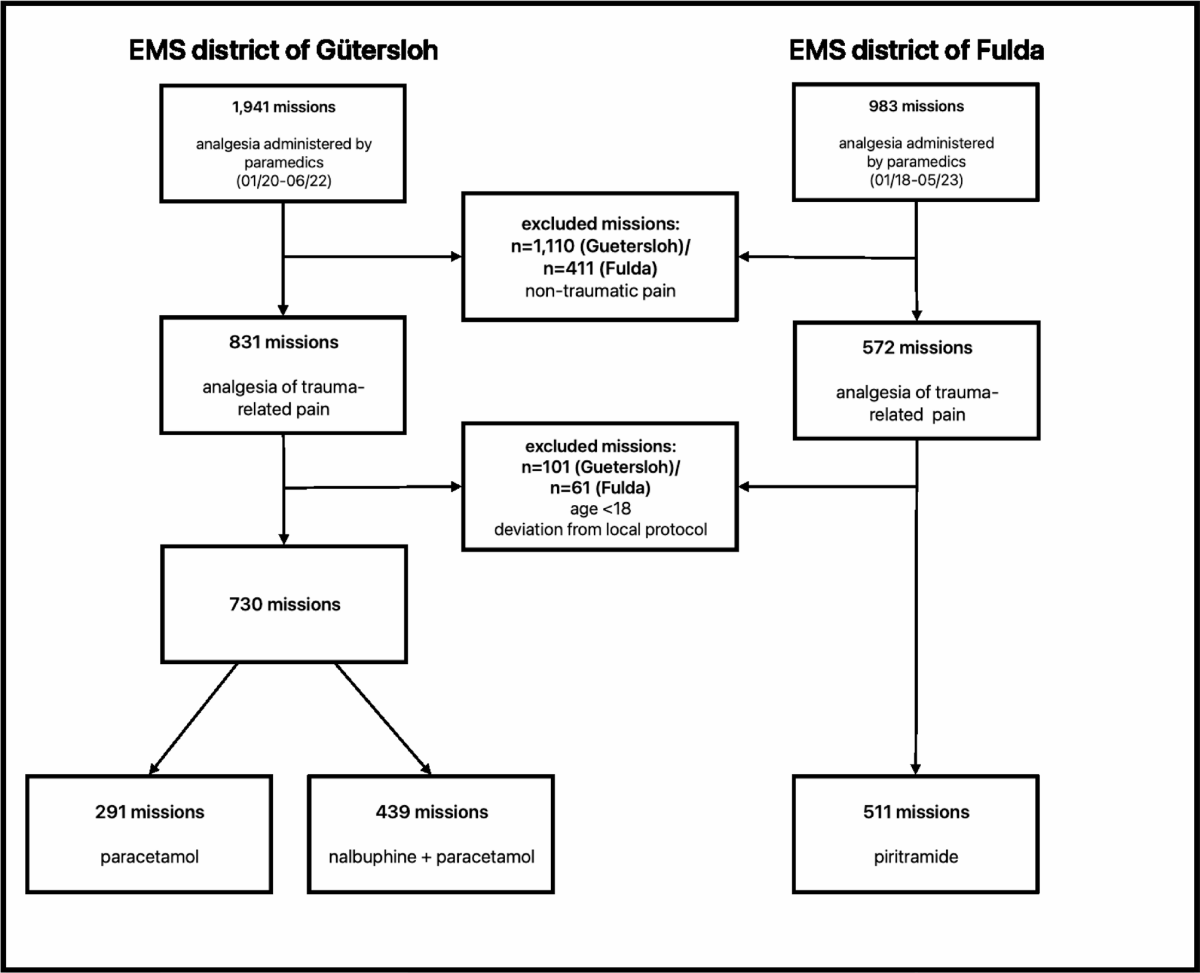
Flowchart of the sample selection process

A total of 2,924 EMS missions involving analgesic therapy by paramedics were recorded across two regions (district of Guetersloh: *n* = 1,941 [66.4%], district of Fulda: *n* = 983 [33.6%]). After applying all the inclusion and exclusion criteria, a total of 1,241 missions (district of Guetersloh: *n* = 730 [58.8%], district of Fulda: *n* = 511 [41.2%]) with analgesia administered for trauma-related pain by paramedics could be evaluated (paracetamol: *n* = 291 [23.4%], nalbuphine plus paracetamol: *n* = 439 [35.4%], piritramide *n* = 511 [41.2%]). Table 1 presents patient characteristics and EMS missions characteristics. Figure 1 illustrates the sample selection process. 

**Table 1:** Patient and mission characteristics

Variables	Total *n* = 1241 [*n* (%)]	Paracetamol *n* = 291 [*n* (%)]	Nalbuphine + paracetamol *n* = 439 [*n* (%)]	Piritramide *n* = 511 [*n* (%)]
Age (years) [Mean ± SD]	60.3 ± 22.7	58.6 ± 22.7	61.0 ± 22.1	60.7 ± 23.1
Female	703 (56.7)	160 (55.0)	257 (58.5)	286 (56.0)
Localization of the injury				
Head/neck	15 (1.2)	13 (4.5)	2 (0.5)	0 (0.0)
Spine	33 (2.7)	15 (5.2)	18 (4.1)	0 (0.0)
Thorax	60 (4.8)	29 (10.0)	12 (2.7)	19 (3.7)
Pelvis	15 (1.2)	11 (3.8)	4 (0.9)	0 (0.0)
Upper extremity	312 (25.1)	44 (15.1)	114 (26.0)	154 (30.1)
Lower extremity	514 (41.4)	86 (29.6)	185 (42.1)	243 (47.6)
Injured several times	20 (1.6)	5 (25.0)	15 (3.4)	0 (0.0)
Not further specified	272 (21.9)	88 (30.2)	89 (20.3)	95 (18.6)
NRS initial [Mean ± SD]	7.9 ± 1.4	6.8 ± 1.4	8.2 ± 1.3	8.3 ± 1.1
NRS hospital admission [Mean ± SD]	4.0 ± 1.8	4.0 ± 1.9	3.6 ± 1.9	4.3 ± 1.5
Change in NRS [Mean ± SD]	3.9 ± 2.0	2.9 ± 2.0	4.6 ± 2.1	4.0 ± 1.5
Total dose of nalbuphine (mg) [Mean ± SD]	14.2 ± 5.9	-	14.2 ± 5.9	-
Total dose of paracetamol (mg) [Mean ± SD]	996.1 ± 45.5	994.3 ± 49.6	997.3 ± 42.7	-
Total dose of piritramide (mg) [Mean ± SD]	7.1 ± 3.5	-	-	7.1 ± 3.5
Occurrence of at least one complication	68 (5.5)	4 (1.4)	34 (7.7)	30 (5.9)
Nausea and vomiting	44 (3.6)	1 (0.3)	19 (4.3)	24 (4.7)
Antiemesis	73 (5.9)	5 (1.7)	41 (9.3)	27 (5.3)
Vigilance reduction	16 (1.3)	1 (0.3)	12 (2.7)	3 (0.6)
Respiratory insufficiency	8 (0.6)	1 (0.3)	4 (0.9)	3 (0.6)
Hemodynamic insufficiency	5 (0.4)	1 (0.3)	1 (0.2)	3 (0.6)
Legend	mg = milligram; NRS = numeric rating scale; SD = standard deviation			

### Primary outcome - effective pain relief

A total of 497 [38.4%] of patients who received analgesic therapy achieved NRS scores ≤ 4 at hospital admission (nalbuphine + paracetamol: *n* = 236 [47.5%]; piritramide: *n* = 142 [28.6%]; paracetamol: *n* = 119 [23.9%]. At hospital admission, the mean NRS score after treatment with nalbuphine + paracetamol was 3.6 ± 1.9, after treatment with piritramide was 4.3 ± 1.5, and after treatment with paracetamol was 4.0 ± 1.9. Table 2 shows the patient characteristics grouped according to the NRS score at hospital admission, while Table 3 shows the number of patients grouped according to the NRS score reduction.

**Table 2:** Patient characteristics grouped according to the Numeric Rating Scale at hospital admission

	**NRS at handover <4**				**NRS at handover 4-5**				**NRS at handover ≥6**			
	Total (n=497)	Nalbuphine + paracetamol (n=236)	Piritramide (n=142)	Paracetamol (n=119)	Total (n=560)	Nalbuphine + paracetamol (n=148)	Piritramide (n=292)	Paracetamol (n=119)	Total (n=200)	Nalbuphine + paracetamol (n=55)	Piritramide (n=89)	Paracetamol (n=55)
Variable	[n (%)]	[n (%)]	[n (%)]	[n (%)]	[n (%)]	[n (%)]	[n (%)]	[n (%)]	[n (%)]	[n (%)]	[n (%)]	[n (%)]
Age in years (MW±SD)	60.0 ± 23.0	60.8 ± 23.0	59.0 ± 23.0	60.1 ± 23.0	60.3 ± 23.0	62.1 ± 23.0	60.0 ± 23.0	58.1 ± 23.0	58.3 ± 23.0	58.9 ± 23.1	59.0 ± 23.0	56.4 ± 23.0
**Initial NRS**												
MW±SD	7.6 ± 1.4	8 ± 1.4	7.8 ± 1.4	6.4 ± 1.4	8.0 ± 1.4	8.3 ± 1.4	8.4 ± 1.4	6.9 ± 1.4	8.5 ± 1.4	8.7 ± 1.4	8.9 ± 1.4	7.7 ± 1.4
**NRS at handover**												
MW±SD	2.3 ± 1.8	2.2 ± 1.8	2.6 ± 1.7	2.3 ± 1.8	4.4 ± 1.8	4.4 ± 1.8	4.4 ± 1.7	4.4 ± 1.8	6.9 ± 1.8	7.2 ± 1.8	6.8 ± 1.7	6.8 ± 1.8
**NRS-change**												
MW±SD	5.2 ± 1.9	5.8 ± 1.9	5.2 ± 1.8	4.1 ± 1.9	3.6 ± 1.9	3.9 ± 1.9	3.9 ± 1.8	2.5 ± 1.9	1.7 ± 1.9	1.7 ± 1.9	2.1 ± 1.9	0.9 ± 2.0
**Applied dose (∑)**												
Nalbuphine (mg) ±SD	14.1 ± 8.3	14.1 ± 8.3			14.0 ± 8.3	14 ± 8.3			15.2 ± 8.3	15.2 ± 8.3		
Paracetamol (mg) ±SD	995.2 ± 45.6	996.6 ± 45.7		992.4 ± 45.6	995.7 ± 45.4	997.3 ± 46.2		993.7 ± 45.4	1000 ± 45.9	1000 ± 46.2		1000 ± 46.1
Piritramide (mg) ±SD	6.6 ± 3.5		6.6 ± 3.5		6.9 ± 3.5		6.9 ± 3.5		8,2 ± 3.5		8.2 ± 3.5	
Female	278 (55.9)	128 (54.2)	77 (54,2)	73 (61.3)	165 (29.5)	94 (63.5)	165 (56.5)	60 (50.4)	109 (54.5)	35 (63.6)	45 (50.6)	28 (50.9)
Nausea/vomiting	17 (3.4)	12 (5.1)	5 (3.5)	0 (0.0)	23 (4.1)	6 (4.0)	16 (5.5)	1 (0.8)	4 (27.3)	1 (1.8)	3 (3.4)	0 (0.0)
Vigilance reduction	8 (1.6)	7 (3.0)	1 (0.7)	0 (0.0)	5 (0.9)	2 (1.4)	2 (0.7)	1 (0.8)	3 (1.5)	3 (5.5)	0 (0.0)	0 (0.0)
Respiratory insufficiency	5 (1.0)	3 (1.3)	1 (0.7)	1 (0.8)	3 (0.5)	1 (0.7)	2 (0.7)	0 (0.0)	0 (0.0)	0 (0.0)	0 (0.0)	0 (0.0)
Hemodynamic insufficiency	3 (0.6)	1 (0.4)	1 (0.7)	1 (0.8)	2 (0.4)	0 (0.0)	2 (0.7)	0 (0.0)	0 (0.0)	0 (0.0)	0 (0.0)	0 (0.0)
**Localization of the injury **												
Head/neck	7 (1.4)	1 (0.4)	0 (0.0)	6 (5.0)	6 (1.1)	1 (0.7)	0 (0.0)	4 (2.5)	3 (1.5)	0 (0.0)	0 (0.0)	3 (5.5)
Spine	20 (4.0)	13 (5.5)	0 (0.0)	7 (5.9)	7 (1.3)	3 (2.0)	0 (0.0)	4 (2.5)	6 (3.0)	2 (3.6)	0 (0.0)	4 (7.3)
Thorax	26 (5.2)	10 (4.2)	7 (4.4)	9 (7.6)	30 (5.4)	2 (1.4)	12 (4.1)	16 (13.4)	5 (2.5)	0 (0.0)	0 (0.0)	5 (9.1)
Pelvis	8 (1.6)	1 (0.4)	0 (0.0)	7 (5.9)	6 (1.1)	2 (1.4)	0 (0.0)	4 (2.5)	1 (0.5)	1 (1.8)	0 (0.0)	0 (0.0)
Upper extremity	105 (21.1)	45 (19.1)	47 (33.1)	13 (10.9)	151 (27.0)	53 (35.8)	80 (27.4)	18 (15.1)	62 (31.0)	16 (29.1)	33 (37.1)	13 (23.6)
Lower extremity	214 (43.1)	112 (47.5)	63 (44.4)	39 (32.8)	235 (42.0)	54 (36.5)	143 (49.0)	38 (31.9)	71 (35.5)	19 (34.5)	42 (47.2)	10 (18.2)
Multiple injuries	11 (2.2)	8 (3.4)	0 (0.0)	3 (2.5)	8 (1.4)	7 (4.7)	0 (0.0)	1 (0.8)	1 (0.5)	0 (0.0)	0 (0.0)	1 (1.8)
Not further specified	106 (21.3)	46 (19.5)	25 (17.6)	35 (29.4)	117 (20.9)	26 (17.6)	57 (19.5)	34 (28.6)	51 (25.5)	17 (30.9)	14 (15.7)	19 (34.5)

**Abbreviations**: mg = milligramm; MW = mean value; NRS = Numeric Rating Scale; SD = standard deviation; ∑ = sum

**Table 3:** Patients grouped according to the NRS score reduction from initial NRS to NRS at hospital admission

Variables	∆NRS < 2 [*n* (%)]	∆NRS = 2 [*n* (%)]	∆NRS = 3 [*n* (%)]	∆NRS = 4 [*n* (%)]	∆NRS > 4 [*n* (%)]
Paracetamol	66 (22.7%)	71 (24.4%)	61 (21.0%)	40 (13.7%)	53 (18.2%)
Nalbuphine + paracetamol	29 (6.6%)	24 (5.5%)	61 (13.9%)	91 (20.7%)	234 (53.5%)
Pritramide	22 (4.3%)	62 (12.1%)	102 (20.0%)	148 (29.0%)	177 (34.6%)
Legend	∆NRS = Difference between initial NRS and NRS at hospital admission				

Table 4 shows the results of the logistic regression analysis for the treatment outcome “NRS at hospital admission ≤ 4”. While treatment with nalbuphine plus paracetamol increased the odds of achieving NRS ≤ 4 at hospital admission (OR: 3.25, 95% CI: 2.46–4.30, *p* < 0.01), treatment with piritramide (OR: 0.31, 95% CI: 0.23–0.41, *p* < 0.01) or paracetamol (OR: 0.32, 95% CI: 0.23–0.45, *p* < 0.01) reduced this chance.

**Table 4:** Results of logistic regression for the outcome numeric rating scale score ≤ 4 at hospital admission

Variables	Odds ratio	95% confidence interval	*p*-value
Age	1.00	1.00‒1.01	0.7565
Sex (female vs. male)	1.03	0.79‒1.33	0.8395
***Initial numeric*** ***rating scale***	***0.66***	***0.60***‒***0.73***	***< 0.0001***
***Nalbuphine + paracetamol vs. piritramide***	***3.25***	***2.46***‒***4.30***	***< 0.0001***
***Piritramide vs. nalbuphine + paracetamol***	***0.31***	***0.23***‒***0.41***	***< 0.0001***
***Paracetamol vs. nalbuphine + paracetamol***	***0.32***	***0.23***‒***0.45***	***< 0.0001***

### Secondary outcome - adverse events

Drug-related complications (nausea/vomiting, antiemetic use, reduced level of consciousness, respiratory or hemodynamic insufficiency) occurred in a total of 68 [5.5%] patients (nalbuphine plus paracetamol: *n* = 34 [7.7%]; piritramide: *n* = 30 [5.9%]; paracetamol: *n* = 4 [1.4%]) (see Table 1). While the comparison of paracetamol vs. nalbuphine plus paracetamol showed lower odds of complications during treatment (OR: 0.17, 95% CI: 0.06–0.49, *p* < 0.01), the comparison of nalbuphine plus paracetamol vs. piritramide showed no evidence for a difference (OR: 0.75, 95% CI: 0.45–1.24, *p* = 0.3). However, a more detailed analysis reveals a higher number of documented antiemetic drug administrations in the nalbuphine group, potentially indicating an increased incidence of nausea and vomiting (nalbuphine plus paracetamol: *n* = 41 [9.3%], piritramide: *n* = 27 [5.3%]).

The results of the logistic regression analysis for the occurrence of at least one complication are shown in Table 5.

**Table 5:** Result of logistic regression for the occurrence of at least one complication

Variable	Odds ratio	95% confidence interval	*p*-value
***Age***	***1.02***	***1.00***‒***1.03***	***0.0109***
Sex (female vs. male)	1.67	0.93‒3.00	0.0863
Piritramide vs. nalbuphine + paracetamol	0.75	0.45‒1.24	0.2607
***Paracetamol vs. nalbuphine + paracetamol***	***0.17***	***0.06***‒***0.49***	***0.001***

## Discussion

### Main findings

This retrospective, multicenter observational study evaluated the efficacy and safety of analgesic therapy for trauma-related pain. In contrast to monotherapy with paracetamol or piritramide, treatment with nalbuphine plus paracetamol increased the likelihood of achieving NRS ≤ 4 on admission to the hospital. Treatment-related complications were rare, mainly related to nausea and/or vomiting, and did not differ between the opioid analgesics used.

### Prehospital analgesia for trauma-related pain

Adequate prehospital analgesia after traumatic injuries is a decisive quality criterion for emergency medical care [14–16]. Despite negative effects associated with the physiological pain response, these patients continue to be inadequately treated with analgesics, often out of concern for potentially life-threatening adverse effects [5, 16–23]. International studies have frequently evaluated the use of fentanyl, morphine and/or (es-)ketamine for prehospital analgesia of trauma-related pain and are therefore also recommended in international guidelines [24–27]. Studies investigating the analgesic effect and safety of piritramide, which is widely used in German-speaking countries, and of a combination of nalbuphine plus paracetamol administered by paramedic are still lacking.

Owing to the changes in the legal basis in Germany, strategies for the implementation of optimal prehospital analgesic therapy by paramedics are still a topic of international controversy and debate. Arguments against piritramide and nalbuphine plus paracetamol often include their lower analgesic potency compared to strong µ-opioid receptor agonists such as fentanyl, as well as the withdrawal symptoms potentially caused by nalbuphine in patients receiving preexisting opioid therapy. However, the advantages of nalbuphine are its favorable pharmacokinetics and a lower rate of life-threatening complications [8]. The present analysis provides evidence for the analgesic efficacy and safety of prehospital analgesia by paramedics using both piritramide and nalbuphine plus paracetamol. The application of nalbuphine plus paracetamol appeared to be particularly suitable for achieving NRS scores ≤ 4 at hospital admission, despite initially comparable pain levels.

There are several possible reasons for this: Firstly, piritramide and nalbuphine have almost identical analgesic potencies of 0.7‒0.75 and 0.7‒1.1 morphine equivalence, respectively, and comparable durations of action of approximately 3–6 h. However, the main differences between the two substances lie in the time to onset of action (piritramide ≤ 16.8 min; nalbuphine ≤ 3 min) and the time from onset of action to peak effect (piritramide ≤ 45 min; nalbuphine approximately 10 min). Owing to these pharmacokinetic peculiarities, it is possible that comparable prehospital analgesia cannot be achieved to the same extent by piritramide during the average durations of an ambulance transport to the hospital [28–30]. Secondly, while an average total dose of 14.2 ± 5.9 mg nalbuphine was administered, a significantly lower total dose of 7.1 ± 3.5 mg piritramide was administered. It is therefore possible, that a comparable analgesic effect could have been achieved by administering increased doses of piritramide, provided that the different pharmacokinetics would not lead to a relevant prolongation of prehospital care time or an increase in complication rate. In this context, the ceiling effect of nalbuphine is an advantage that provides a high degree of safety regarding respiratory complications, which makes slow titration unnecessary [31, 32].

Furthermore, while paracetamol, as known from the literature, was not shown to be as effective as a single substance in the present study [1, 3, 33, 34], data concerning its use as a co-analgesic show inconsistent results [35]. While an opioid-sparing effect of paracetamol could not be proven in emergency rooms [36], studies in perioperative medicine have revealed both a reduction in the need for opioids and a lack of an additive effect [37]. The additive administration of paracetamol in the present analysis could have enhanced the analgesic effect of nalbuphine. Despite the heterogeneous data, this additional effect may also manifest in combined analgesic therapy with piritramide and paracetamol.

### Possible effects of prehospital analgesia with nalbuphine on the clinical course

Although the present results demonstrate that analgesic therapy with nalbuphine plus paracetamol is effective and safe in trauma patients, its pharmacodynamic properties as a partial µ-antagonist/κ-agonist regularly raise concerns about potential impairments and interactions in the context of further medical care. These discussions include possible interactions with subsequent µ-agonists (e.g., morphine, fentanyl, and sufentanil), for example, in the emergency department or during further surgical treatment in the hospital. Although nalbuphine exhibits partial µ-antagonism with up to 1/3 the potency of the µ-antagonist naloxone, the data regarding the possibility of administering µ-agonists following nalbuphine administration are inconsistent: While some studies report no adverse effects during treatment in the emergency department [36, 38], others suggest a need for increased doses of µ-agonists post-nalbuphine without any increase in drug-related complications (e.g. hypotension, bradycardia, hypoventilation) [39, 40]. Whether and, if so, to what extent prehospital analgesia with nalbuphine as a partial µ-antagonist could influence the general anesthesia required for surgical treatment has not yet been sufficiently evaluated. However, there is the possibility of displacing the partial µ-antagonist nalbuphine through competitive agonism with a µ-agonist. In this context, remifentanil appears particularly suitable because of its short duration of action and short context-sensitive half-life, with minimal risk of accumulation and remorphinization after the effects of nalbuphine have subsided. From a clinical perspective, there are currently no significant concerns regarding the use of nalbuphine in prehospital emergency care, provided that subsequent treating physicians are informed about its administration and can adjust their choice of analgesics accordingly. Future studies should evaluate the implications of prehospital nalbuphine administration for in-hospital and perioperative care.

### Limitations

This study has several limitations that should be considered when interpreting the findings. First of all, the retrospective observational design limits the ability to infer casual relationships and introduces potential selection and documentation bias. Systematic differences between the observed regions and the lack of available data on exact mission and transport times may act as potential confounding factors. Although both study regions are comparable in terms of their rural character and EMS structures, variations in prehospital duration and transport durations may still have influenced the degree of pain relief observed at hospital admission and could have influenced the observed differences between the analgesics used. Furthermore, the comparison of the piritramide and nalbuphine plus paracetamol groups was based only on patients with an initial NRS score of ≥ 7, due to the limitations of the matched-pairs design and the resulting missings, limiting the exploration of outcomes in patients with lower initial pain scores. While the use of the NRS for evaluating pain intensity is controversial because of its subjectivity, it allows a simple and practical evaluation of the indication and effectiveness of analgesia, which is why it is recommended by professional societies and widely used in (pre-)hospital settings. Furthermore, the effectiveness and complications of the analgesic concepts investigated could not be assessed over time, for example, in the emergency department or during surgery. Despite this, the results provide important insights into the development of treatment options for trauma-related pain that are associated with minimal complications and high efficacy.

## Conclusions

In contrast to monotherapy with paracetamol or piritramide, combination therapy with nalbuphine plus paracetamol for trauma-related pain increased the likelihood of achieving an NRS score ≤ 4 upon hospital arrival. Drug-related complications were rare, predominantly involving nausea and/or vomiting, and did not differ when comparing the opioid analgesics used. Future studies should evaluate the effects of prehospital administration of nalbuphine by paramedics in in-hospital and perioperative settings.

## Data Availability

The datasets used and/or analyzed during the current study are available from the corresponding author upon reasonable request.

## Declarations

### Prior presentations

Parts of the present work have been presented as a poster at the German Interdisciplinary Emergency Congress (DINK 2025) 21.03. − 21.03.2025, Koblenz, Germany.

## Abbreviations

EMS: Emergency medical services
BtMG: Germany’s narcotics act (Gesetz über den Verkehr mit Betäubungsmitteln; Betäubungsmittelgesetz)
NRS: Numeric rating scale
GCS: Glasgow coma scale
mg/kg: Milligrams per kilogram body weight
95% CI: 95% confidence interval
OR: Odds ratio

## References

[1] Häske D, Böttiger BW, Bouillon B, Fischer M, Gaier G, Gliwitzky B, et al. Analgesie bei Traumapatienten in der Notfallmedizin. Anaesthesist. 2020;69(2):137–48. 10.1007/s00101-020-00735-4 32002561 10.1007/s00101-020-00735-4

[2] Bischof F, Kaczmarek C. Analgesie beim Erwachsenen in der prähospitalen Notfallmedizin. Notfallmedizin Up2date. 2024;19(02):165–87. 10.1055/a-2056-3301

[3] Michael M, Hossfeld B, Häske D, Bohn A, Bernhard M. Analgesie, Sedierung und anästhesie in der notfallmedizin. Anästh Notfallmedizin. 2020;61:51–65. 10.19224/ai2020.051

[4] Deutsche Gesellschaft für Unfallchirurgie e.V. & Deutsche Gesellschaft für Orthopädie und Unfallchirurgie e.v. Polytrauma/Schwerverletzten-Behandlung S3-Leitlinie. 2022;(4.0). https://register.awmf.org/assets/guidelines/187-023l_S3_Polytrauma-Schwerverletzten-Behandlung_2023-06.pdf

[5] Albrecht E, Taffe P, Yersin B, Schoettker P, Decosterd I, Hugli O. Undertreatment of acute pain (oligoanalgesia) and medical practice variation in prehospital analgesia of adult trauma patients: a 10 year retrospective study. Br J Anaesth. 2013;110(1):96–106. 10.1093/bja/aes355 23059961 10.1093/bja/aes355

[6] Bundesamt für Justiz. BtMG. [cited 2024 Dec 28]. Available from: https://www.gesetze-im-internet.de/btmg_1981/

[7] Hinrichs M, Weyland A, Bantel C. Piritramid. Schmerz. 2017;31(4):345–52. 10.1007/s00482-017-0197-y 28265754 10.1007/s00482-017-0197-y

[8] Deslandes M, Deicke M, Grannemann JJ, Hinkelbein J, Hoyer A, Kalmbach M, et al. Prähospitale Analgesie mit Nalbuphin und Paracetamol im Vergleich zu Piritramid durch Notfallsanitäter*innen – eine multizentrische Observationsstudie. Anaesthesiol. 2024;73(9):583–90. 10.1007/s00101-024-01449-7 10.1007/s00101-024-01449-7 PMC11358200 39177686

[9] Deslandes M, Deicke M, Grannemann JJ, Hinkelbein J, Hoyer A, Kalmbach M, et al. Effectiveness and safety of prehospital analgesia with Nalbuphine and Paracetamol versus morphine by paramedics - an observational study. Scand J Trauma Resusc Emerg Med. 2024;32(1):41. 10.1186/s13049-024-01215-z 38730453 10.1186/s13049-024-01215-z PMC11084095

[10] Zeng Z, Lu J, Shu C, Chen Y, Guo T, Wu Q, ping, et al. A comparision of Nalbuphine with morphine for analgesic effects and safety: Meta-Analysis of randomized controlled trials. Sci Rep. 2015;5:10927. 10.1038/srep10927 26039709 10.1038/srep10927 PMC4454168

[11] Gal TJ, DiFazio CA, Moscicki J. Analgesic and respiratory depressant activity of nalbuphine: a comparison with morphine. Anesthesiology. 1982;57(5):367–74. 10.1097/00000542-198211000-00004 6814301 10.1097/00000542-198211000-00004

[12] Romagnoli A, Keats AS. Ceiling effect for respiratory depression by Nalbuphine. Clin Pharmacol Ther. 1980;27(4):478–85. 10.1038/clpt.1980.67 7357806 10.1038/clpt.1980.67

[13] Hossfeld B, Holsträter S, Bernhard M, Lampl L, Helm M, Kulla M. Prähospitale Analgesie beim Erwachsenen – Schmerzerfassung und Therapieoptionen. AINS - Anästhesiol · Intensivmed · Notfallmed · Schmerzther. 2016;51(02):84–96. 10.1055/s-0042-101466 26949902 10.1055/s-0042-101466

[14] Kumle B, Wilke P, Koppert W, Kumle K, Gries A. Schmerztherapie in der Notfallmedizin: Fokus Notaufnahme. Anaesthesist. 2013;62(11):902–13. 10.1007/s00101-013-2247-x 24173544 10.1007/s00101-013-2247-x

[15] Matthes G, Trentzsch H, Wölfl CG, Paffrath T, Flohe S, Schweigkofler U, et al. [Essential measures for prehospital treatment of severely injured patients: the trauma care bundle]. Unfallchirurg. 2015;118(8):652–6. 10.1007/s00113-015-0042-7 26160129 10.1007/s00113-015-0042-7

[16] Stork B, Hofmann-Kiefer K. Analgesie in der Notfallmedizin. Notf Rettungsmedizin. 2008;11(6):427–38. 10.1007/s10049-008-1093-x

[17] Vassiliadis J, Hitos K, Hill CT. Factors influencing prehospital and emergency department analgesia administration to patients with femoral neck fractures. Emerg Med Fremantle WA. 2002;14(3):261–6. 10.1046/j.1442-2026.2002.00341.x 10.1046/j.1442-2026.2002.00341.x 12487043

[18] Bakkelund KE, Sundland E, Moen S, Vangberg G, Mellesmo S, Klepstad P. Undertreatment of pain in the prehospital setting: a comparison between trauma patients and patients with chest pain. Eur J Emerg Med Off J Eur Soc Emerg Med. 2013;20(6):428–30. 10.1097/MEJ.0b013e32835c9fa3 10.1097/MEJ.0b013e32835c9fa3 23242076

[19] Bounes V, Barniol C, Minville V, Houze-Cerfon CH, Ducassé JL. Predictors of pain relief and adverse events in patients receiving opioids in a prehospital setting. Am J Emerg Med. 2011;29(5):512–7. 10.1016/j.ajem.2009.12.005 20825821 10.1016/j.ajem.2009.12.005

[20] Moller JC, Ballnus S, Kohl M, Gopel W, Barthel M, Kruger U, et al. Evaluation of the performance of general emergency physicians in pediatric emergencies: obstructive airway diseases, seizures, and trauma. Pediatr Emerg Care. 2002;18(6):424–8. 10.1097/00006565-200212000-00005 12488835 10.1097/00006565-200212000-00005

[21] Pierik JGJ, IJzerman MJ, Gaakeer MI, Berben SA, van Eenennaam FL, van Vugt AB, et al. Pain management in the emergency chain: the use and effectiveness of pain management in patients with acute musculoskeletal pain. Pain Med Malden Mass. 2015;16(5):970–84. 10.1111/pme.12668 10.1111/pme.12668 25546003

[22] Ricard-Hibon A, Chollet C, Saada S, Loridant B, Marty J. A quality control program for acute pain management in out-of-hospital critical care medicine. Ann Emerg Med. 1999;34(6):738–44. 10577403 10.1016/s0196-0644(99)70099-5

[23] Stalnikowicz R, Mahamid R, Kaspi S, Brezis M. Undertreatment of acute pain in the emergency department: a challenge. Int J Qual Health Care J Int Soc Qual Health Care. 2005;17(2):173–6. 10.1093/intqhc/mzi022 10.1093/intqhc/mzi022 15723816

[24] Jennings PA, Cameron P, Bernard S, Walker T, Jolley D, Fitzgerald M, et al. Morphine and ketamine is superior to morphine alone for out-of-hospital trauma analgesia: a randomized controlled trial. Ann Emerg Med. 2012;59(6):497–503. 10.1016/j.annemergmed.2011.11.012 22243959 10.1016/j.annemergmed.2011.11.012

[25] Galinski M, Dolveck F, Borron SW, Tual L, Van Laer V, Lardeur JY, et al. A randomized, double-blind study comparing morphine with Fentanyl in prehospital analgesia. Am J Emerg Med. 2005;23(2):114–9. 10.1016/j.ajem.2004.03.010 15765326 10.1016/j.ajem.2004.03.010

[26] Gausche-Hill M, Brown KM, Oliver ZJ, Sasson C, Dayan PS, Eschmann NM, et al. An Evidence-based guideline for prehospital analgesia in trauma. Prehosp Emerg Care. 2014;18(Suppl 1):25–34. 10.3109/10903127.2013.844873 24279813 10.3109/10903127.2013.844873

[27] Godwin SA, Burton JH, Gerardo CJ, Hatten BW, Mace SE, Silvers SM, et al. Clinical policy: procedural sedation and analgesia in the emergency department. Ann Emerg Med. 2014;63(2):247–e25818. 10.1016/j.annemergmed.2013.10.015 24438649 10.1016/j.annemergmed.2013.10.015

[28] Strickmann B, Deicke M, Hoyer A, Kobiella A, Jansen G. Effectiveness and safety of prehospital analgesia including Nalbuphine and Paracetamol by paramedics: an observational study. Minerva Anestesiol. 2023;89(12):1105–14. 10.23736/S0375-9393.23.17537-7 38019174 10.23736/S0375-9393.23.17537-7

[29] Gnirke A, Krautz T, Oehmke L, Marung H. Qualitätssicherung bei der Anwendung von Standardarbeitsanweisungen (SAA) und erweiterten Versorgungsmaßnahmen in der Rettungsdienst-Kooperation in Schleswig-Holstein (RKiSH) gGmbH. Notf Rettungsmedizin. 2023 Apr 28 [cited 2024 Dec 28]; Available from: 10.1007/s10049-023-01150-z

[30] Schütte F, Fürst N, Szyprons A, Schmitz S, Weber B, Käser B et al. Analyse des Leistungsniveaus im Rettungsdienst für die Jahre 2020 und 2021. 2024. 10.60850/bericht-m345

[31] Fehn K. Analgesie mit opioidhaltigen Arzneimitteln durch Notfallsanitäter unter der Geltung des Notfallsanitätergesetzes. Medizinrecht. 2017;35(6):453–9. 10.1007/s00350-017-4620-2

[32] Vijayvargiya M, Panchal S, Asawale K, Desai A. Oligoanalgesia in the emergency setting – An Indian review. J Clin Orthop Trauma. 2021;18:38–43. 10.1016/j.jcot.2021.03.025 33996447 10.1016/j.jcot.2021.03.025 PMC8091043

[33] Feth M, Knapp J, Hossfeld B. Analgesie bei Sportverletzungen. Notf Rettungsmedizin. 2022;25(7):464–72. 10.1007/s10049-022-01082-0

[34] Kiavialaitis GE, Müller S, Braun J, Rössler J, Spahn DR, Stein P, et al. Clinical practice of pre-hospital analgesia: an observational study of 20,978 missions in Switzerland. Am J Emerg Med. 2020;38(11):2318–23. 10.1016/j.ajem.2019.10.033 31785972 10.1016/j.ajem.2019.10.033

[35] Galinski M, Ruscev M, Gonzalez G, Kavas J, Ameur L, Biens D, et al. Prevalence and management of acute pain in prehospital emergency medicine. Prehosp Emerg Care. 2010;14(3):334–9. 10.3109/10903121003760218 20507221 10.3109/10903121003760218

[36] Chambers JA, Guly HR. Prehospital intravenous Nalbuphine administered by paramedics. Resuscitation. 1994;27(2):153–8. 8029537 10.1016/0300-9572(94)90008-6

[37] Friesgaard KD, Riddervold IS, Kirkegaard H, Christensen EF, Nikolajsen L. Acute pain in the prehospital setting: a register-based study of 41.241 patients. Scand J Trauma Resusc Emerg Med. 2018;26(1):53. 10.1186/s13049-018-0521-2 29970130 10.1186/s13049-018-0521-2 PMC6029421

[38] Stene JK, Stofberg L, Macdonald G, Myers RA, Ramzy A. Nalbuphine Analgesia in the Prehospita Setting. Therapeutics. 10.1016/0735-6757(88)90109-x 3178962

[39] Schultz-Machata AM, Becke K, Weiss M. Nalbuphin in der Kinderanästhesie. Anaesthesist. 2014;63(2):135–43. 10.1007/s00101-014-2293-z 24504192 10.1007/s00101-014-2293-z

[40] Houlihan KP, Mitchell RG, Flapan AD, Steedman DJ. Excessive morphine requirements after pre-hospital Nalbuphine analgesia. J Accid Emerg Med. 1999;16(1):29. 10.1136/emj.16.1.29 9918283 10.1136/emj.16.1.29 PMC1343250

